# Phase I/IIa trial of androgen deprivation therapy, external beam radiotherapy, and stereotactic body radiotherapy boost for high-risk prostate cancer (ADEBAR)

**DOI:** 10.1186/s13014-020-01665-6

**Published:** 2020-10-08

**Authors:** Yeon Joo Kim, Hanjong Ahn, Choung-Soo Kim, Young Seok Kim

**Affiliations:** 1 Department of Radiation Oncology, Asan Medical Center, University of Ulsan, College of Medicine, 88, Olympic-ro 43-gil, Songpa-gu, Seoul, 05505 Republic of Korea; 2 Department of Urology, Asan Medical Center, University of Ulsan, College of Medicine, Seoul, Republic of Korea

**Keywords:** Prostate neoplasms, Radiotherapy, Radiosurgery, Toxicity, Quality of life

## Abstract

**Background:**

To evaluate the clinical outcomes of combination of androgen deprivation therapy (ADT), whole pelvic radiotherapy (WPRT), and stereotactic body radiotherapy (SBRT) boost in high-risk prostate cancer patients.

**Methods:**

This prospective phase I/IIa study was conducted between 2016 and 2017. Following WPRT of 44 Gy in 20 fractions, patients were randomized to two boost doses, 18 Gy and 21 Gy, in 3 fractions using the Cyberknife system. Primary endpoints were incidences of acute toxicities and short-term biochemical recurrence-free survival (BCRFS). Secondary endpoints included late toxicities and short-term clinical progression-free survival (CPFS).

**Results:**

A total of 26 patients were enrolled. Twelve patients received a boost dose of 18 Gy, and the rest received 21 Gy. The Median follow-up duration was 35 months. There were no grade ≥ 3 genitourinary (GU) or gastrointestinal (GI) toxicities. Sixty-one and 4% of patients experienced grade 1–2 acute GU and GI toxicities, respectively. There were 12% late grade 1–2 GU toxicities and 8% late grade 1–2 GI toxicities. Patient-reported outcomes of urinary symptoms were aggravated after WPRT and SBRT boost. However, they resolved at 1 month and returned to the baseline level at 4 months. Three-year BCRFS was 88.1%, and CPFS was 92.3%.

**Conclusions:**

The present study protocol demonstrated that the combination of ADT, WPRT, and SBRT boosts for high-risk prostate cancer is safe and feasible, and may reduce total treatment time to 5 weeks. Boost dose of 21 Gy in 3 fractions seems appropriate.

**Trial registration:**

ClinicalTrials.gov, ID; NCT03322020 - Retrospectively registered on 26 October 2017.

## Background

For patients affected by intermediate- and high-risk prostate cancer, dose-escalated external beam radiotherapy (EBRT) with a dose range of 76 to 80 Gy has demonstrated improved tumor control with low rates of gastrointestinal (GI) and genitourinary (GU) toxicities [1, 2]. These dose-escalated regimens are delivered in conventional fractionation schemes (1.8–2 Gy/fraction) with a treatment duration of 8–9 weeks. Because prostate cancer has a low alpha-beta ratio compared to adjacent normal organs [3], hypofractionation is a feasible strategy for improving clinical outcomes and shortening treatment time. There is increasing use of high-dose-rate brachytherapy (HDRB) boost combined with EBRT, and the results are promising [4, 5]. However, brachytherapy is invasive. There have been attempts to replace brachytherapy boost with noninvasive stereotactic body radiotherapy (SBRT) boost using CyberKnife, as it may have comparable dose distribution as HDR brachytherapy [6].

While several retrospective studies have explored the feasibility of the CyberKnife boost, they included patients in different risk groups and treated patients with various dose regimens. In addition, radiation fields and androgen deprivation therapy schedules (18–21 Gy/2–3 fractions) also varied [7–12]. A 5-year analysis of a prospective phase II CKNO-PRO trial that assessed CyberKnife boost (18 Gy/3 fractions) followed by conventional EBRT to prostate and seminal vesicles in intermediate-risk prostate cancer was recently published [12]. All patients did not receive androgen deprivation therapy (ADT). The study demonstrated favorable toxicity and quality of life profiles as well as good efficacy results. Unlike the CKNO-PRO trial, the present Androgen Deprivation Therapy, External Beam Radiotherapy and Stereotactic Body Radiotherapy Boost for High-risk Prostate Cancer (ADEBAR) study was designed to target patients with high-risk prostate cancer treated with ADT and whole pelvic radiotherapy (WPRT). The study also evaluated clinical outcomes of two boost dose regimens (18 Gy/3 fractions and 21 Gy/3 fractions) followed by WPRT (44 Gy/20 fractions) to explore appropriate dose regimens for CyberKnife boost.

The primary aim of this phase I/IIa study is to prospectively evaluate acute toxicities in patients with high-risk prostate cancer who received combination therapy of ADT, WPRT, and two different CyberKnife boost regimens. Secondary endpoints included late toxicities and short-term biochemical recurrence-free survival (BCRFS) as well as clinical progression-free survival (CPFS).

## Methods

The present ADEBAR study was approved by the institutional review board and registered on clinicaltrials.gov (NCT03322020). Written informed consent was obtained from all enrolled patients.

**Fig. 1:**
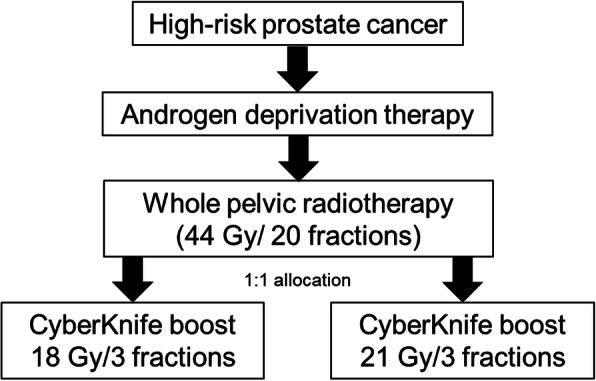
Treatment for ADEBAR trial

Patients were eligible for this trial if they had pathologically confirmed high-risk prostate cancer according to the current National Comprehensive Center Network (NCCN) guidelines (http://www.nccn.org), Eastern Cooperative Oncology Group performance status of 0–1 and adequate laboratory results based on a test performed within 6 months before enrollment according to the institutional protocol for the study [13]. Patients with pelvic lymph node or distant metastasis were excluded after pelvic magnetic resonance imaging (MRI) and bone scan. As shown in Fig. 1, all enrolled patients first received ADT. Generally, long-term ADT is recommended for high-risk patients [14, 15], and individual physicians determine the total period of ADT. Administration of luteinizing hormone-releasing hormone agonists and/or antiandrogens was allowed.

Three to six months after starting ADT, WPRT (44 Gy/20 fractions) was initiated. The dose of this mild hypofractionated WPRT is thought to be equivalent to the dose of standard fractionation (46 Gy/23 fractions). Without increasing bowel toxicity, the regimen might improve tumor control of prostate cancer so that it is routine practice to high risk group in our center. A detailed description of the institutional protocol for radiotherapy was introduced in a previous publication [13]. A week before computed tomography (CT) for treatment planning, three gold intra-prostatic fiducial markers were inserted for CyberKnife tracking. Simulation CT scans with a slice thickness of 2.5 mm were obtained with empty bladders and rectal balloons in place for reproducibility. Gross target volume (GTV) includes whole prostate glands, involved extraprostatic tissues, and any suspicious involved seminal vesicles. Clinical target volume (CTV) included regional nodal areas including an obturator, external/internal iliac, and presacral lymphatic areas. Planning target volume (PTV) was an expansion of CTV margins by 5 mm, except for the posterior 3 mm margin for rectum sparing. PTV must be irradiated with ≥95% of the prescription dose. All patients received intensity-modulated radiotherapy (IMRT). Dose constraints for organs-at-risks (OAR) were as follows: 1) bowel (small and large), 30% of entire bowel volume must not receive more than 40 Gy; 2) rectum, 60% of volume must receive ≤40 Gy; 3) bladder, 35% of bladder volume must receive ≤45 Gy; 4) femoral heads, 15% of femoral head volume must receive < 35 Gy. Daily cone-beam CT was used for image guidance.

CyberKnife boost was administered right after the end of WPRT and treated every other day. For CyberKnife boost planning, a non-enhanced simulation CT with 1.25 mm slices was obtained after enema for bowel preparation without the use of a rectal balloon. Patients were in a supine position with a vacuum cushion and an ankle pillow with an empty bladder. Foley catheters were inserted to delineate the urethra. Boost volume was identical to the GTV of WPRT delineation, whereas the PTV margin was 3 mm in all directions. The urethra, rectum, and bladder were also included in OAR. PTV must receive ≥80 and < 120% of the prescribed dose. OAR dose constraints are as follows: 1) rectum, D_1cc_ (dose delivered to a 1 cm^3^ volume) < 85% of the prescription dose and D_max_ (maximum dose) < 100% of prescription dose; 2) bladder and urethra, D_1cc_ < 100% of the prescription dose and D_max_ < 110% of prescription dose. Patients were randomized into two groups (18 Gy versus 21 Gy in 3 fractions) with a 1:1 allocation. The dose regimens were designed to achieve the same equivalent dose in 2 Gy per fraction (EQD_2_) of conventional dose-escalated EBRT with 78 Gy in 38 fractions. An α/β ratio constant is needed to calculate EQD_2_. This represents the dose at which cellular death rates with linear and quadratic components are equivalent. An estimated α/β ratio for normal tissue is 3, while that for prostate cancer is 1.5 [3]. Normal tissue EQD_2_ for conventional dose is 78 Gy, which is similar to that for SBRT boost dose of 18 Gy (78.2 Gy) and lower than that for a boost regimen of 21 Gy (87.8 Gy). The EQD_2_ values for prostate cancer are 78, 85.1, and 97.5 Gy with conventional regimens and 18 and 21 Gy for boost therapies. Patients in the 18 Gy arm received the same EQD_2_ for adjacent organs, while prostate cancer irradiated 109% EQD_2_ of conventional EBRT, and patients in the 21 Gy group received 113 and 125% EQD_2_ of conventional EBRT for normal tissue and tumor, respectively.

The present study was planned as an exploratory feasibility study, therefore, a total of 15 patients in each dose regimen were considered as an appropriate sample size. Primary endpoints of the ADEBAR trial were incidences of acute toxicities, defined as events occurring within three months after treatment. Acute toxicities were evaluated according to the Common Toxicity Criteria for Adverse Events (CTCAE) v 4.03 and patient-reported outcomes (PROs). PROs included the overactive bladder symptom score (OABSS) and International Prostate Symptom Score (IPSS). PROs were achieved in 5-time points as follows: before WPRT as a baseline, after WPRT, after CyberKnife boost, 1 month after radiotherapy, and 4 months after radiotherapy. The sum of frequency [2], urgency [4], and nocturia [7] of the IPSS questionnaire was defined as the storage symptoms score, while the sum of emptying [1], intermittency [3], weak stream [5], and hesitancy [6] was defined as the voiding symptom score.

Secondary endpoints were short-term (3-year) BCRFS, CPFS and late toxicities. Biochemical recurrence (BCR) is defined as the Phoenix consensus definition of a rise in PSA level by 2 ng/mL or more above the nadir value or clinical progression. Clinical progression was defined as a visible gross lesion found on imaging studies including MRI or bone scan. The biopsy was not routinely necessary for confirmation of recurrence. Late toxicity was evaluated using the Late Effects of Normal Tissues scoring system, short-term CPFS.

During ADT, patients had follow-up visits to the department of urology every three months. Radiation oncologists collected patient data every week during radiotherapy. After radiotherapy, patients visited the department of radiation oncology after one month and every three months for the first two years thereafter. If there was no evidence of recurrence after two years, patients came to the clinic every six months for five years and annually thereafter. Follow-up evaluations include toxicities, PSA, and testosterone levels. If clinical recurrence was suspected, imaging studies such as MRI and/or bone scans were considered.

Incidence of toxicities, OABSS scores, and IPSS scores of patients in the 18 Gy arm and 21 Gy arm were compared by Mann Whitney U-test due to the small study population. A *p*-value of less than 0.05 was considered statistically significant. The Kaplan-Meier method was used to estimate survival. All statistical analyses were performed using SPSS version 21.0.

## Results

We enrolled a total of 30 patients. However, four of them were excluded due to the followings; (1) withdrawal of consent on radiotherapy (*n* = 2), (2) aggravated liver cirrhosis (*n* = 1), (3) diagnosis of bone metastasis. As a result, a total of 26 patients were enrolled between March 2016 and December 2017. The characteristics of the patients are listed in Table 1. Patients were all high-risk groups, and most of them (96%) were classified into a very high-risk group according to NCCN guidelines. Among them, 12 patients received CyberKnife boost dose of 18 Gy, and 14 patients were treated with 21 Gy. Median follow-up duration was 35 months (range, 19–43) from the end of radiotherapy.

**Table 1:** Patient characteristics

Characteristic	Total (***n*** = 26)	18 Gy (***n*** = 12)	21 Gy (***n*** = 14)	p-value
	No. of patients (%)			
Median age (range)	74 years (54–84)	74 years (66–80)	73 years (54–84)	0.860
T stage				0.297
2b	1 (4)	0 (0)	1 (7)	
2c	2 (8)	0 (0)	2 (14)	
3a	14 (54)	7 (58)	7 (50)	
3b	5 (19)	3 (25)	2 (14)	
4	4 (15)	2 (17)	2 (14)	
Grade group				0.462
1	1 (4)	0 (0)	1 (7)	
2	2 (8)	2 (17)	0 (0)	
3	2 (8)	1 (8)	1 (7)	
4	8 (30)	4 (34)	4 (29)	
5	13 (50)	5 (42)	8 (57)	
Initial PSA (ng/mL)				0.252
< 10	5 (19)	1 (8)	4 (29)	
10–20	3 (12)	1 (8)	2 (14)	
> 20	18 (69)	10 (83)	8 (57)	
Risk group				0.781
High	1 (4)	0 (0)	1 (7)	
Very high	25 (96)	12 (100)	13 (93)	
Median duration of ADT, months (range)	25 (9–35)	26 (20–34)	25 (9–35)	0.742
Baseline OABSS (0–15)				0.781
0–5 (mild)	23 (88)	11 (92)	12 (86)	
6–11 (moderate)	2 (8)	1 (8)	1 (7)	
12–15 (severe)	1 (4)	0 (0)	1 (7)	
Baseline IPSS (0–35)				0.231
1–7 (mild)	7 (27)	5 (42)	2 (14)	
8–19 (moderate)	16 (62)	6 (50)	10 (72)	
20–35 (severe)	3 (11)	1 (8)	2 (14)	
Baseline QoL				0.036
Delighted	5 (19)	5 (42)	0 (0)	
Pleased	5 (19)	1 (8)	4 (29)	
Mostly satisfied	4 (15)	2 (17)	2 (14)	
Mixed	7 (27)	2 (17)	5 (36)	
Mostly dissatisfied	3 (12)	1 (8)	2 (14)	
Unhappy	2 (8)	1 (8)	1 (7)	
Terrible	0 (0)	0 (0)	0 (0)	

*ADT* androgen deprivation therapy, *IPSS* International Prostate Symptom Score, *OABSS* overactive bladder symptom score, *PSA* prostate-specific antigen, *QoL* quality of life

Baseline PRO data were available in all 26 patients (Table 1). Results of the OABSS questionnaire indicated that the majority of patients (88%) had mild urinary symptoms with an average value of 3.2 (range, 1–5). According to the IPSS questionnaire, 27% of patients had mild symptoms with an average score of 5 (range, 4–6), and the majority of patients (62%) demonstrated moderate symptoms with an average score of 12.8 (range, 8–19). There was no statistically significant difference between 18 Gy and 21 Gy arms, except in baseline quality of life, which included more patients who answered “delighted” in 18 Gy arm.

**Fig. 2:**
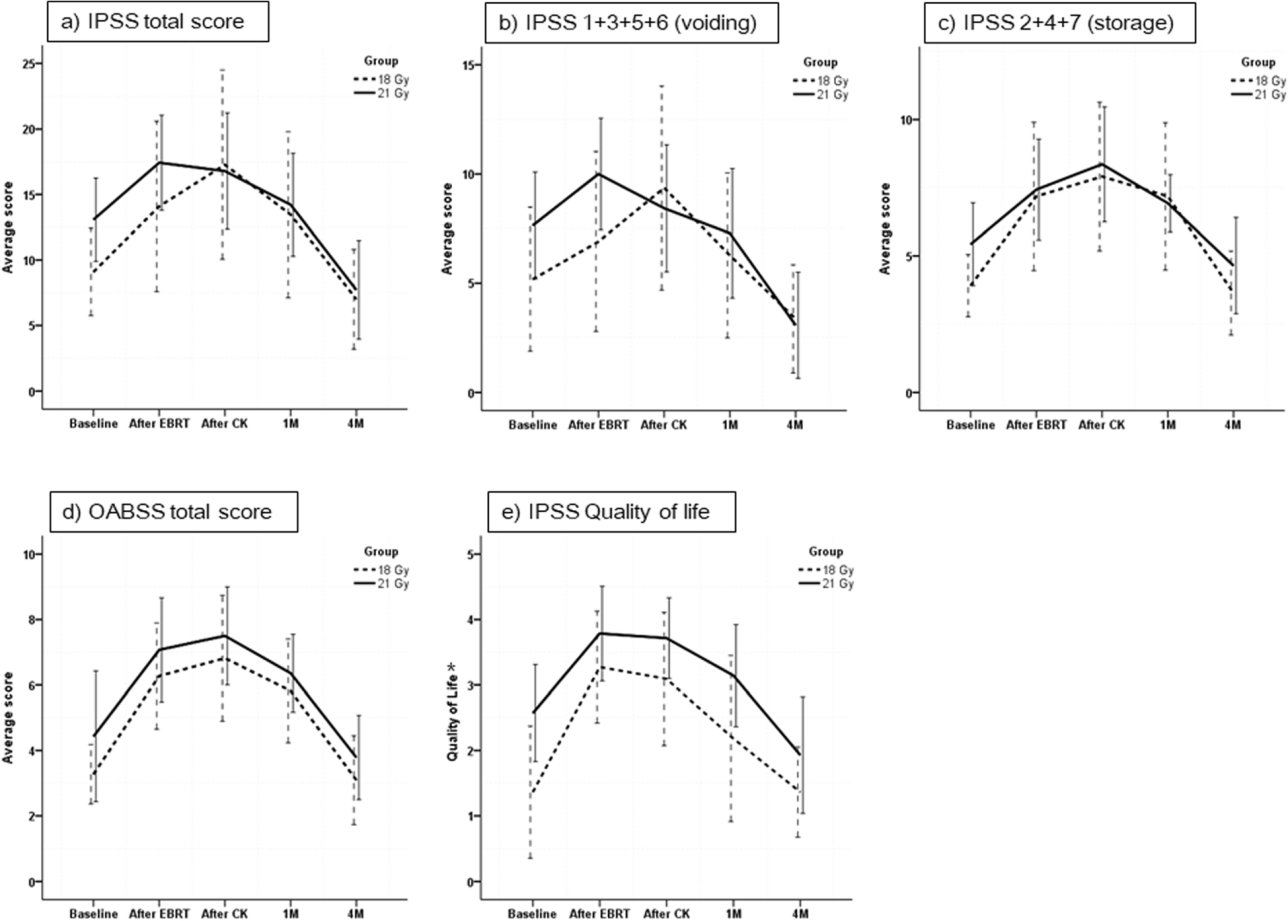
Results of patient-reported outcomes utilizing International Prostate Symptom Score (IPSS) and Overactive Bladder Symptom Score (OABSS) questionnaires in 25 patients. **a** IPSS total score, **b** IPSS voiding score, **c** IPSS storage score, **d** OABSS total score, and **e** IPSS Quality of Life [^*^Numbers on y-axis represent Delighted (0), Pleased (1), Mostly satisfied (2), Mixed (3), Mostly dissatisfied (4), and unhappy (5)]. Abbreviation = *M* months

Figure 2 demonstrates changes in symptom scores at 5-time points (baseline, after WPRT, after CyberKnife boost, 1 month after radiotherapy, and 4 months after radiotherapy) in 25 patients. One patient did not answer the questionnaire at 1 month and a 4-month follow-up, so his data were excluded. IPSS total scores (Fig. 2a) were worse after WPRT, which persisted after the CyberKnife boost. However, symptoms improved after 1 month and eventually became similar or slightly better at 4 months compared to baseline. This trend could be seen in the IPSS voiding score, storage scores, and OABSS (Fig. 2b-d). Quality of life index from IPSS was also aggravated after WPRT and CyberKnife boost, but improved at 1 month and returned to baseline status at 4 months. Both groups showed the same trend in score changes.

Acute and late toxicities measured by physicians were listed in Table 2. There were no grade 3 or higher toxicities. Grade 1 and 2 acute GU toxicities occurred in 38% (*n* = 10) and 23% (*n* = 6) of total patients, respectively. Nocturia, the most common symptom, occurred in 12 cases and was followed in frequency by dysuria (*n* = 5) and urgency (*n* = 4). Two cases of late grade 1 nocturia and one case of grade 2 urgency were reported, but all resolved within 1 year. Acute GI toxicity occurred in one patient with anal hemorrhage in the 21 Gy arm, while 2 patients in the 18 Gy group experienced late GI toxicities of grade 1 fecal incontinence and grade 2 proctitis (rectal bleeding). Grade 2 proctitis resolved after hip bath without any medication, and the other late GI toxicities also improved spontaneously within 1 year.

**Table 2:** Summary of acute and late toxicities

			18 Gy (n = 12)	21 Gy (n = 14)	Total (n = 26)
GU	Acute	Grade 1	6 (50%)	4 (29%)	10 (38%)
		Grade 2	3 (25%)	3 (21%)	6 (23%)
	Late	Grade 1	1 (8%)	1 (7%)	2 (8%)
		Grade 2	0	1 (7%)	1 (4%)
GI	Acute	Grade 1	0	1 (7%)	1 (4%)
	Late	Grade 1	1 (8%)	0	1 (4%)
		Grade 2	1 (8%)	0	1 (4%)

*GI* gastrointestinal, *GU* genitourinary

**Fig. 3:**
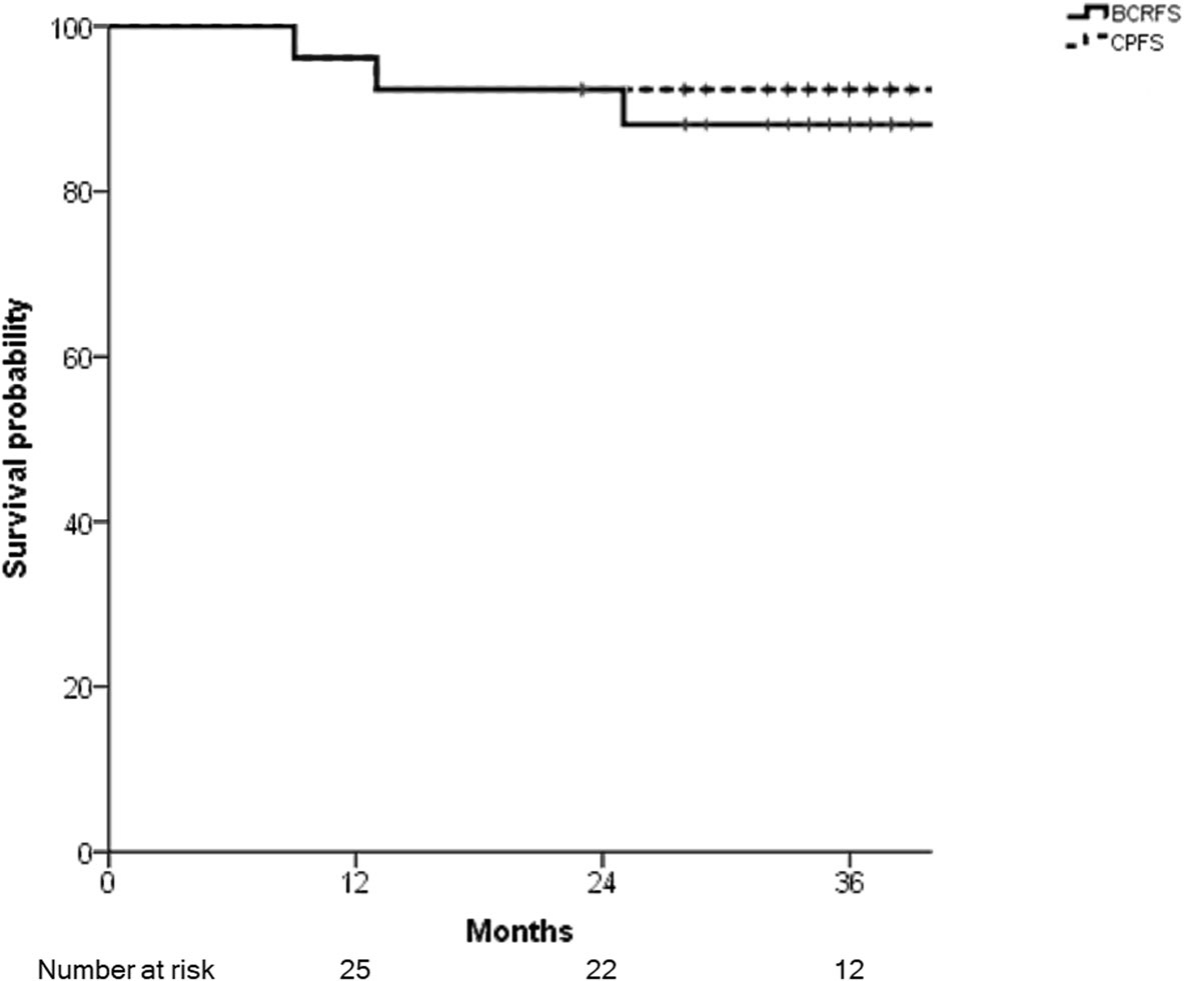
Graphs of biochemical recurrence-free survival (BCRFS) and clinical progression-free survival (CPFS)

Three-year BCRFS and CPFS were also evaluated in 26 patients (Fig. 3). Three-year BCRFS was 88.1%, and 3-year CPFS was 92.3%. Three patients experienced BCR, and two of them also had simultaneous clinical progression. All three patients were very high-risk group having multiple high-risk factors, such as T stage of 3, grade group ≥4, and initial PSA over 30 ng/mL. They received ADT for 28 months, and two received CyberKnife boost dose of 18 Gy. Two patients with clinical progression had relatively short progression-free intervals of 9 and 13 months, while one with only BCR had 25 months of disease-free interval.

Most patients enrolled in the present study received ADT over 1 year. Usual recovery of testosterone occurs slowly over 1–2 years [16]. The median follow-up time of 35 months (range of 19 to 43 months) is insufficient to determine the exact long-term tumor control rate after testosterone recovery. Testosterone levels were checked regularly to evaluate the survival of patients who achieved testosterone recovery, defined as testosterone > 2.5 ng/mL. Eight of 26 patients achieved testosterone recovery, and all 8 patients have no evidence of disease over 23 months after radiotherapy.

## Discussion

The ADEBAR protocol was well tolerated with no reported grade 3 or higher toxicities in both groups using CyberKnife boost dose of 18 Gy and 21 Gy in 3 fractions. PRO results indicate that most patients experienced aggravated urinary symptoms and worse quality of life during radiotherapy. However, symptoms resolved and returned to the baseline status at 4 months. This correlated with physician-reported outcomes, which demonstrated a higher incidence of acute grade 1–2 GU toxicities (61%) compared to only 12% of late toxicities. Two boost dose regimens (18 Gy versus 21 Gy) demonstrated similar acute toxicities so that boost dose of 21 Gy also seems tolerable for CyberKnife boost after WPRT.

Several studies evaluated SBRT boost using various modalities after EBRT, and their results are summarized in Table 3 [7–12, 17, 18]. Except for two studies, the majority of studies utilized CyberKnife as a modality for the SBRT boost. Joseph et al. [17] used the HDRB boost (19.5 Gy/3 fractions) and compared the results to multiple conventional EBRT regimens (66 Gy, 70 Gy, and 74 Gy). Although long-term absolute serious adverse events only occurred in 3 patients, the rate of urethral stricture was 12.7% at a median follow-up of 7.4 years in the HDRB group compared with 0.8–3.8% in other EBRT groups. The most common reason for urethral stricture is iatrogenic from urethral manipulation (45%), and HDRB is an invasive procedure that might increase the incidence of urethral stricture [19]. HDRB might be replaced by EBRT while retaining its high-dose-rate effect. Pryor et al. [18] evaluated a gantry-based linear accelerator for SBRT boost and reported a 2-year cumulative incidence of grade 2 or more GU toxicities of 24.9%. This result was slightly higher than that reported by other studies, except Anwar et al. [9], which reported a rate of 1.4–14% (Table 3). Anwar et al. demonstrated 27% late grade 2 GU toxicities, similar to Pryor et al. Both studies used a regimen with 19–21 Gy doses in 2 fractions. These results suggest that 18–21 Gy in 3 fractions is better than a 2 fraction regimen in terms of late GU toxicities.

**Table 3:** Summary of previous studies on stereotactic body radiotherapy boost after external beam radiotherapy

Author (Y)	Design	No.	Risk group (%)	EBRT (Gy/fx)	WPRT rate	Boost modality (%)	Boost (Gy/fx)	Administration of ADT/duration (M)	Median f/u (M)	Late toxicities	BCRFS
Pasquier (2020) [12]	P	76	I (100)	46/23	None	CK (79) Gantry-based (21)	18/3	None	62	5Y cumulative incidence G ≥ 2: 1.4% (GU), 9.3% (GI)	5Y 87.4%
Joseph (2019) [17]	P	237	I (14)	46/23	None	HDRB (100)	19.5/3	48%/6	126^a^	Absolute 1.3% of SAE	10Y LRFS^a^ 97.8%
			H (86)					52%/18			
										7.4Y urethral stricture: 12.7%	
Pryor (2019) [18]	P	135	I (76)	46/23	8%	Gantry-based (100)	19 or 20/2	36%/ ≤6	24	2Y cumulative incidence G ≥ 2: 24.9% (GU), 4.5% (GI)	2Y 98.6%
			H (24)					18%/> 6		Two G3 (GU), one G3 (GI)	
Kim (2017) [11]	R	39	I (51)	45/25	100%	CK (100)	21/3	None	54	G2 10.3% (GU), 12.8% (GI)	5Y 94.7%
			H (49)							No G ≥ 3	
Mercado (2016) [7]	R	108	L (4)	45–50.4/25–28	None	CK (100)	19.5/3	64%/median 6 M	53	2Y cumulative incidence (moderate to big by EPIC): 13.7% (GU), 5% (GI)	3Y 100%(I), 89.8%(H)
			I (42)								
			H (54),								
Anwar (2016) [9]	P	48	I (29)	45/25	100%	CK (100)	19 or 21/2	88%/median 6 M	43	G2 27% (GU), None (GI)	5Y 90%
			H (71)							One G3 case	
Lin (2014) [10]	R	41	H (100)	45/25	100%	CK (100)	21/3	100%/long-term	42	G2 3–11% (GU), None (GI)	4Y 91.9%
										No G ≥ 3	
Katz (2014) [8]	R	45	H (100)	45/25	100%	CK (100)	18–21/3	62%/NA	69	GU: G2 2.3%, G3 2.3%	6Y 69%
										GI: G2 13.3%, No G3	
Present	P	26	H (100)	44/20	100%	CK (100)	18 or 21/3	100%/median 25 M	35	No G ≥ 2 (GU), G2 4% (GI)	3Y 88.1%

*ADT* androgen deprivation therapy, *BCRFS* biochemical recurrence-free survival, *CK* CyberKnife, *EBRT* external beam radiotherapy, *EPIC* expanded prostate cancer index composite, *f/u* follow-up, *fx* fractions, *G* grade, *GI* gastrointestinal, *GU* genitourinary, *H* high, *HDRB* high-dose-rate brachytherapy, *I* intermediate, *L* low, *M* months, *LRFS* local recurrence-free survival, *NA* not available, *No*. number, *P* prospective, *R* retrospective, *SAE* Serious adverse event, *WPRT* whole pelvic radiotherapy, *Y* year

^a^ Local progression was defined as recurrent prostatic mass diagnosed by digital rectal examination or imaging techniques

Studies with more than 50 patients enrolled did not routinely provide WPRT. Some studies included patients in a high-risk group. Although there is no definite evidence from randomized trials regarding the administration of WPRT to high-risk groups [20], there are persistent efforts to reveal the value of WPRT for high-risk prostate cancer. A recent non-randomized prospective study included 812 patients with localized prostate cancer treated with EBRT and a single 15 Gy HDRB boost. Compared to prostate only EBRT, BCRFS was greater when WPRT was used, particularly in high-risk patients [21]. WPRT increased both prevalence and cumulative incidence of acute GI and GU toxicities, but there was no difference in late toxicities. In the present ADEBAR stud, with a median follow-up of 35 months, there was only one grade 2 late toxicity (4%). Other studies that administered WPRT and 3 fractions for boost dose regimen demonstrated 5–14% of grade ≥ 2 late GU toxicities and 0–13% of GI toxicities, indicating the safety of WPRT.

Unlike other studies, the present study utilized a mild hypofractionated regimen (2.2 Gy/fraction) for WPRT. Despite the dose regimen, toxicities were minimal. This was attributed to the use of a rectal balloon for prostate immobilization and rectal sparing, daily use of cone-beam CT, and IMRT planning. The urethra was contoured, and 100% or overdose irradiation to the urethra was avoided. Although acute toxicities according to OABSS and IPSS questionnaires increased soon after WPRT and CyberKnife boost, symptoms stabilized to the baseline level at 4 months. The present protocol reduces total treatment time from 8 weeks to 5 weeks. It is also convenient for patients and reduces the cost of medical services.

Tumor control effects were also favorable with a 3-year BCRFS of 88.1% and CPFS of 92.3%. These rates are comparable to the outcomes of two retrospective studies that enrolled high-risk patients only [8, 10] and reported a 3-year BCRFS of 91.9 and 74% (estimated from survival graph). Outcomes of Lin et al. [10] demonstrate better biochemical control, but their study only included 22% very high-risk patients compared to 96% in the present study. The follow-up period of 35 months in the ADEBAR study is too short to assess biochemical control endpoints in the setting of long-term ADT. As a preliminary report, we assessed tumor control and recovery of testosterone levels, and there was no recurrence in 8 patients who had testosterone recovery. This suggests favorable long-term BCRFS with further follow-up in the ADEBAR setting. Follow-up is ongoing to assess longer-term survival rates and late toxicities.

There is growing evidence of SBRT boost following WPRT in high-risk groups. However, to our knowledge, there is no phase III randomized control study comparing conventional dose-escalated EBRT versus SBRT boost. The ASCENDE-RT study highlighted the importance of radiation dose for intermediate and high-risk prostate cancer for improving BCRFS in the brachytherapy boost arm compared to the dose-escalated EBRT arm in the setting of WPRT [22]. Because prostate cancer has been reported to have greater sensitivity to higher doses per fraction than nearby organs [23], HDRB or hypofractionation including SBRT has been actively adopted with promising results [24].

The present prospective ADEBAR study is limited by a small number of patients and short follow-up period for evaluating biochemical control. The durations of ADT were different by each urologist as there was no exact study guideline for ADT prescription. Also, the long-term results of the PROs were not available. However, the main goal of the present phase I/IIa study was to evaluate feasibility in patients treated with ADT and WPRT (44 Gy/20 fractions) followed by SBRT boost doses of 18 Gy and 21 Gy in 3 fractions. Acute toxicities were confined to grades 1–2, PROs were relieved at 4 months, and there were minimal late toxicities confined to grade 1. Because two boost dose regimens were well tolerated, we conclude that 21 Gy in 3 fractions is appropriate for future utilization. We are planning a phase III randomized study comparing dose-escalated EBRT (78 Gy/39 fractions) and SBRT boost (21 Gy/3 fractions) after WPRT (44 Gy/20 fractions). This dose regimen has EQD_2_ of 97.5 Gy, which is 19.5 Gy higher than the conventional regimen. According to a previous meta-analysis, a 2.6% improvement of BCRFS is expected for each additional Gy [25].

## Conclusions

The present study suggests that the combination of ADT, WPRT, and SBRT boost for high-risk prostate cancer is safe and feasible, and its outcomes should be evaluated in a phase III randomized trial. SBRT boost dose of 21 Gy in 3 fractions might be appropriate with the CyberKnife platform.

## Data Availability

The datasets analyzed during the current study are available from the corresponding author on reasonable request.

## Abbreviations

ADT: Androgen deprivation therapy
ADEBAR: Androgen Deprivation Therapy, External Beam Radiotherapy and Stereotactic Body Radiotherapy Boost for High-risk Prostate Cancer
BCR: Biochemical recurrence
BCRFS: Biochemical recurrence-free survival
CPFS: Clinical progression-free survival
CTV: Clinical target volume
CTCAE: Common Toxicity Criteria for Adverse Events
CT: Computed tomography
EQD2: Equivalent dose in 2 Gy per fraction
EBRT: External beam radiotherapy
GI: Gastrointestinal
GU: Genitourinary
GTV: Gross target volume
HDRB: High-dose-rate brachytherapy
IMRT: Intensity-modulated radiotherapy
IPSS: International Prostate Symptom Score
MRI: Magnetic resonance imaging
NCCN: National Comprehensive Center Network
OAR: Organs-at-risks
OABSS: Overactive bladder symptom score
PROs: Patient-reported outcomes
PTV: Planning target volume
SBRT: Stereotactic body radiotherapy
WPRT: Whole pelvic radiotherapy
